# Microporous Carbon Nanoparticles for Lithium–Sulfur Batteries

**DOI:** 10.3390/nano10102012

**Published:** 2020-10-12

**Authors:** Hui-Ju Kang, Gazi A. K. M. Rafiqul Bari, Tae-Gyu Lee, Tamal Tahsin Khan, Jae-Woo Park, Hyun Jin Hwang, Sung Yong Cho, Young-Si Jun

**Affiliations:** 1 Department of Advanced Chemicals & Engineering, Chonnam National University, 77 Yongbong-ro, Buk-gu, Gwangju 61186, Korea; gmlwn120@gmail.com (H.-J.K.); grafiqulbari@gmail.com (G.A.K.M.R.B.); dlxorb007@gmail.com (T.-G.L.); jaewoopark0218@gmail.com (J.-W.P.); 2 Department of Materials Science and Engineering, Chonnam National University, 77 Yongbong-ro, Buk-gu, Gwangju 61186, Korea; tamalche15@gmail.com; 3 School of Chemical Engineering, Chonnam National University, Gwangju 61186, Korea; wgguswls@gmail.com; 4 Department of Environment and Energy Engineering, Chonnam National University, 77 Yongbong-ro, Buk-gu, Gwangju 61186, Korea

**Keywords:** lithium–sulfur batteries, microporous, eutectic salt, amorphous carbon, energy storage

## Abstract

Rechargeable lithium–sulfur batteries (LSBs) are emerging as some of the most promising next-generation battery alternatives to state-of-the-art lithium-ion batteries (LIBs) due to their high gravimetric energy density, being inexpensive, and having an abundance of elemental sulfur (S_8_). However, one main, well-known drawback of LSBs is the so-called polysulfide shuttling, where the polysulfide dissolves into organic electrolytes from sulfur host materials. Numerous studies have shown the ability of porous carbon as a sulfur host material. Porous carbon can significantly impede polysulfide shuttling and mitigate the insulating passivation layers, such as Li_2_S, owing to its intrinsic high electrical conductivity. This work suggests a scalable and straightforward one-step synthesis method to prepare a unique interconnected microporous and mesoporous carbon framework via salt templating with a eutectic mixture of LiI and KI at 800 °C in an inert atmosphere. The synthesis step used environmentally friendly water as a washing solvent to remove salt from the carbon–salt mixture. When employed as a sulfur host material, the electrode exhibited an excellent capacity of 780 mAh g^−1^ at 500 mA g^−1^ and a sulfur loading mass of 2 mg cm^−2^ with a minor capacity loss of 0.36% per cycle for 100 cycles. This synthesis method of a unique porous carbon structure could provide a new avenue for the development of an electrode with a high retention capacity and high accommodated sulfur for electrochemical energy storage applications.

## 1. Introduction

Efficient energy storage demand is growing to a greater extent. The steady market of portable devices, vehicle electrification, aviation, robotics, marine, military, and grid-scale energy reserve strategies has inspired advanced research into energy stockpile mechanisms [1]. Lithium-ion batteries (LIBs) play a significant role in the existing commercial world. There is always a burden of cost in the production of LIBs as Ni, Mn, and Co are used as cathode materials ($ < 100/kWh). Safety is a concern for LIBs, and 10–15% of electrical energy is lost as heat during LIB discharge. In large power packs for hybrid electric vehicles, a lack of heat management could cause an explosion [2]. Furthermore, for LIBs, it is hard to achieve an energy density of ~240 Wh kg^−1^ [3]. The alternative energy storage system of lithium–sulfur batteries (LSBs) is a potential claimant from various perspectives. The theoretical energy density for lithium–sulfur batteries is 2600 Wh kg^−1^ or 2800 Wh L^−1^ [4]. The earth-abundant and inexpensive (0.02 $ g^−1^) sourcing of sulfur has put forward the potentiality of LSBs [5]. Elemental sulfur is one of the most significant byproducts of the large petroleum industry; moreover, sulfur is benign from an environmental perspective [6]. From a chemical point of view, sulfur, as an active material, causes a multistep redox reaction in charge and discharge mechanisms [7]. Each sulfur atom provides two electrons; thus, LSBs retain their high specific capacity and energy density, where the active material of LIBs generates a single electron [8,9].

Despite all this potentiality and these benefits, lithium–sulfur batteries face some technical challenges. Elemental sulfur serves as an active material, which undergoes reduction via the formation of a series of polysulfides (Li_2_S_n_; 8 ≥ *n* ≥ 2) [10,11]—the charge/discharge mechanism of LSBs, the formation of Li_2_S during discharge, and elemental S during charge. Sulfur and discharge products possess very low electrical conductivity. During discharge, long-chain polysulfides ions are formed in the cathode, which are soluble in the electrolyte at the time of operation. Soluble polysulfides diffuse through the separator to the anode (lithium metal), where they are reduced to insoluble Li_2_S_2_ or Li_2_S and passivate the anode [12,13]. Here, deposited insoluble Li_2_S/Li_2_S_2_ passivation impede electrical contact and ion transport. Simultaneously, pursuant loss of active materials by the dissolution of Li_2_S (*n* = 4–8) on the electrolyte leads to variable internal resistance and the rise of parasitic mass.

Moreover, the volume expansion of sulfur occurs during the charge/discharge process, consisting of the flare-up fading of its capacity, a poor cycle life, low specific capacity, and the low energy efficiency of the overall battery system [14,15]. Efforts have intensified in addressing the glitch. An effective strategy consists of confining the polysulfides on a cathode structure to improve performance by reducing the diffusion of dissolution products on the anode. Encompassing conductive additives, porous supporting materials on active materials bolster the sulfur’s electronic and ionic conductivity [16,17]. Additionally, the low solubility of the polymer/solid electrolyte for polysulfides to attenuate the dissolution from cathode materials has been introduced [18]. The use of additive materials to encapsulate the anode metal’s active surface to safeguard against Li_2_S passivation has been considered [17]. Different research attempts have presented different strategies to mitigate these challenges. A carbon/sulfur nanocomposite was used to make the bimodal pore structure of carbon. The small mesopore contained a significant portion of sulfur and stamped out polysulfides’ diffusion into the electrolyte. Additionally, sizable interconnected pores assisted the quick transport of solvated Li during the charge/discharge operation [19]. Graphene and sulfur composites have provided high electrical conductivity of 1820 S cm^−1^, accommodating ample space for volume expansion due to their significant ability to confine polysulfides [20]. Such a conductive skeleton improves the initial performance or fast capacity loss; however, it cannot prevent polysulfides’ ultimate dissolution. The interaction between nonpolar carbon materials and polar hydrophilic lithium polysulfides is fragile, causing the shaky confinement ability of polysulfides [21,22]. It is incredible to note that the defective nature of amorphous carbon provides a substantial effect on electrochemical performance. Polysulfides prefer to bond on defective sites (vacant monosite: adsorption energy of Li_2_S_4_: −0.62 eV; Li_2_S: −3.35 eV) where the polysulfides are trapped, boosted, and reduced into lithium sulfide [7]. A functionalized carbon nanotube (CNT) was coated with a uniform ZnS nanoparticle working as a sulfur host by chemical adsorption, which mitigated the shuttle effect [23]. Additionally, porous carbon derived from an Al-based metal–organic framework composite and a carbon nanotube with 50% sulfur reduced the diffusion of polysulfide by encapsulating elemental sulfur in the pore structure of CNT@PC (porous carbon) [24]. Ordered mesoporous carbon with a bimodal pore size of 3 and 6 nm was utilized as a sulfur host [25]. An effort to lock the sulfur on a nitrogen-doped porous carbon embedded with cobalt and a composite with an interconnected carbon nanotube helped to improve electronic conductivity and bolstered the capability rate [26]. A porous carbon nanofiber functionalized with a carbonyl group assembled on nickel has been synthesized. The carbonyl group eased polysulfide diffusion due to its chemical affinity, and its inner porous space of carbon reinforcement provided high sulfur loading and volume expansion [27]. The sulfur precursor, polyacrylonitrile (PAN), and carbon nanotube were used to fabricate a freestanding composite thin film by an electrospinning method followed by an annealing process. The pre-addition of the sulfur spurred the sulfurization of PAN and generated a porous structure on the CNT. Interconnected porous CNT promotes lithium-ion diffusion and electrical conductivity [28]. A study compared and examined polysulfide’s confinement ability by introducing sulfur into distinct types of carbon structures such as graphene, flake graphite, and CNTs [29]. Graphene/S showed the best cycle stability and reversibility at a low current density. On the other hand, CNT/S provided good capacity at high charging and discharging conditions. A functional group containing oxygen on the carbon structure provided efficient hindrance of the polysulfide’s dissolution and good cycle stability. Even though CNT provided the efficient enhancement of sulfur utilization and hindrance as a consequence of the polysulfide, it could not fully control the polysulfide’s disclosure to the electrolyte. Moreover, CNT had a small aspect ratio, poor uniformity, limited production, and low mechanical strength and structural stability. In the functionalized CNT, it was difficult to control the doping amount [30].

This study focused on the synthesis of microporous amorphous carbon using the LiI/KI eutectic salts mixture with the high-temperature solvent method from the nature-abundant biomass of glucose. Salt templating synthesis is usually used to synthesize the formation of pore morphology on the structure of what is administered in the formation of macropores and mesopores [31]. By adopting the concept of eutectic low-temperature melting, this research proceeds with pore control engineering. Additionally, several studies on aluminum or its alloys in corrosion science concluded that molten salts (especially halogen-based) are corrosive to metal, leaving significant challenges in applications when it comes to using those salts at high temperatures. This corrosion science gives us the assumption that facile reactive templating synthetic methods might be achieved by combining relevant eutectic mixtures, which are reactive to carbonaceous species, leaving functional groups on the surface. The most magnificent conception of this study, lithium-ion and iodide, strongly solubilizes glucose on the salt medium by disengaging the hydrogen bonds. Simultaneously, the large size and strong nucleophilic ability of iodide influenced the formulation of a different mode of primary intermediates before initiating the polycondensation of carbon [32]. Fusion salt formed smaller pores and a larger surface area [32]. The choice of cation size and counterion (LiI/KI) helped to obtain a controlled hierarchical micropore structure. The LiI/KI eutectic salts mixture has a unique role, acting as an on-site pore-forming proxy within the carbonization process. LiI/KI showed the path of forming an effective hierarchical microporous carbon structure that is a significant host for S. Electrochemical discharge products of polysulfides remained in the cathode and minimized the shuttling effect by maintaining optimal clog on the structure and in the trapping of S. Moreover, the mesopore induced the ionic transportation of Li-ion. Compact surface contact and the ascension of the degree of aromatic condensation helped to improve electrical conductivity, ultimately enhancing the LSBs performance.

## 2. Materials and Methods

### 2.1. Materials

Glucose (98%, JUNSEI Co., Tokyo, Japan), lithium iodide (99%, Sigma-Aldrich Inc., Darmstadt, Germany), potassium iodide (99.0%, Sigma-Aldrich Inc., Darmstadt, Germany), carbon disulfide (99%, Sigma-Aldrich Inc., Darmstadt, Germany), lithium bis(trifluoromethylsulfonyl)imide (LiTFSI, 99.95%, Sigma-Aldrich Inc., Darmstadt, Germany), 1,2-dimethoxy ethane (DME, 99.5%, Sigma-Aldrich Inc., Darmstadt, Germany), 1,3-dioxolane (DOL, 99.8%, Sigma-Aldrich Co., Saint Louis, MO, USA), lithium nitrate (99.99%, Sigma-Aldrich Co., Saint Louis, MO, USA), polypropylene separator (MTI (Massachusetts Technologies Incorporation) Co., Richmond, CA, USA), Li metal (99.9%, Alfa Aesar Co., Haverhill, MA, USA), polyvinylidene fluoride (PVdF, MTI Korea Co., Richmond, CA, USA), Super P (Timcal Co., Bodio, Switzerland), *N*-methyl pyrrolidone (99.0%, DAEJUNG Co., Siheung, Korea), and C-coated aluminum foil (MTI Co., CA, USA) were used in the study.

### 2.2. Synthesis of GL-I-800

Glucose (0.5 g), lithium iodide (2.9 g), and potassium iodide (2.1 g) were mixed using a mortar and pestle. The weight ratio of the glucose and eutectic salts mixture (LiI/KI) was 1:10. After that, the combination of glucose and salts was heated at 800 °C (heating rate: 2.3 °C min^−1^) for 5 h under a nitrogen atmosphere in a tube furnace. After cooling to room temperature, the resulting black and white chunk was dispersed into distilled water and stirred at 100 °C in an oil bath overnight to remove the salts. The dispersion was filtered using a Stericup filter and washed with triple distilled water. The residue was then dried in an oven at 90 °C. The dried powder was transferred into a glass vial and dry in an oven at 120 °C for one more day. The synthesized powder is denoted as GL-I-800 (glucose carbonized with eutectic salt LiI/KI at 800 °C). The glucose without eutectic salt mixture of LiI/KI was also calcined in similar conditions and was labeled as GL-800 (glucose carbonized at 800 °C).

### 2.3. Characterization

The FT-IR (Fourier transform infrared spectrometer) spectrum was characterized by a Fourier transform infrared spectrometer (JASCO FT-IR 4100, JASCO Inc., Easton, MD, USA). X-ray photoelectron spectroscopy (XPS) spectra were obtained on a Thermo Scientific, Waltham, MA, USA Kα XPS spectrometer with a monochromatized, microfocused Al Kα line source. The crystal structure of all studied samples was obtained with the Rigaku D/max Ultima III instrument with Cu Kα radiation (*λ* = 0.154056 Å) (Rigaku, TX, USA). Raman spectroscopy was conducted on an Aberration-corrected Czerny–Turner monochromator (NRS-5100, JASCO Inc., Easton MD, USA) at 532.13 nm. The surface morphologies of the synthesized materials were studied by field-emission scanning electron microscopy (FE-SEM, HITACHI, SU-70, Fukuoka, Japan) and high-resolution transmission electron microscopy (HR-TEM, TECNAI F20 UT, FEI CO., Hillsboro, OR, USA). Energy-dispersive spectroscopy (EDS) elemental mapping was performed on a field-emission scanning electron microscopy (FE-SEM, HITACHI, EDX-200, Fukuoka, Japan). Nitrogen adsorption and desorption isotherms were determined by nitrogen physisorption at 77 K on a Micromeritics ASAP 2020 analyzer (Norcross, GA, USA). The samples were degassed at 150 °C for 12 h under vacuum before analysis.

### 2.4. Electrochemical Measurements

#### 2.4.1. Preparation of the Porous Carbon and Sulfur (C+S) Composite

A 140.4 mg amount of sulfur powder was dissolved in CS_2_. The solubility of elemental sulfur (orthorhombic S_8_) in CS_2_ at 25 °C was 34.8 wt %. The GL-I-800 powder was then added to the S/CS_2_ solution and sonicated at 60 °C for 1 h to remove the CS_2_ solvent. CS_2_ was added into the remnant and this process was repeated two more times under the same condition for the perfect infiltration of sulfur into the carbon structure. The synthesized C+S composite was dried in a vacuum oven at 60 °C for 24 h. CS_2_ is easily volatilized and dangerous in case of inhalation. Therefore, this process must be carried out in a fume hood. It can be confirmed by the result of X-ray diffraction (XRD).

#### 2.4.2. Preparation of Electrode

The electrode used for the electrochemical test was prepared by a slurry casting method. In brief, the slurry containing the C+S composite, Super P as a conductive additive, and polyvinylidene fluoride (PVdF) as a binder in a weight ratio of 80:10:10 was sonicated in *N*-methyl pyrrolidone for 1 h to disperse contents and stirred overnight. The slurry was then uniformly cast onto C-coated aluminum foil (MTI Co., Richamond, CA, USA) and dried in a vacuum oven at 60 °C for 24 h.

#### 2.4.3. Electrochemical Characterization

The prepared cathode materials’ electrochemical performance was examined using a CR-2032 coin-type cell (MTI Co., Richamond, CA, USA) with a lithium foil anode (MTI Co., Richamond, CA, USA) and a separator (MTI Co., Richamond, CA, USA). The electrolyte contained 1 M lithium bis(trifluoromethane sulfonyl) imide (LiTFSI) in a mixed solution of 1,3-dioxolane (DOL) and 1,2-dimethoxyethane (DME) (1:1 by volume). All cells were assembled in a glove box (Koreakiyon Co., Seoul, Korea) filled with an argon atmosphere. The galvanostatic tests were carried out on the WMPG1000S (WonATech, Seoul, Korea) within a voltage range of 1.8–2.6 V vs. Li/Li^+^ and a current density of 500 mA g^−1^. The mass loading of sulfur in each electrode was 2–6 mg cm^−2^. All the specific capacity was based on the mass of sulfur. Cyclic voltammetry (CV) at a scan rate of 0.1 mV/s within a voltage range of 1.0–3.0 V vs. Li/Li^+^ and electrochemical impedance spectra (EIS) measurements a frequency range 3 × 10^5^ –5 × 10^−2^ Hz were conducted on a Biologic VSP (Biologic Co., Grenoble, France) potentiostat.

## 3. Results

**Scheme 1:**
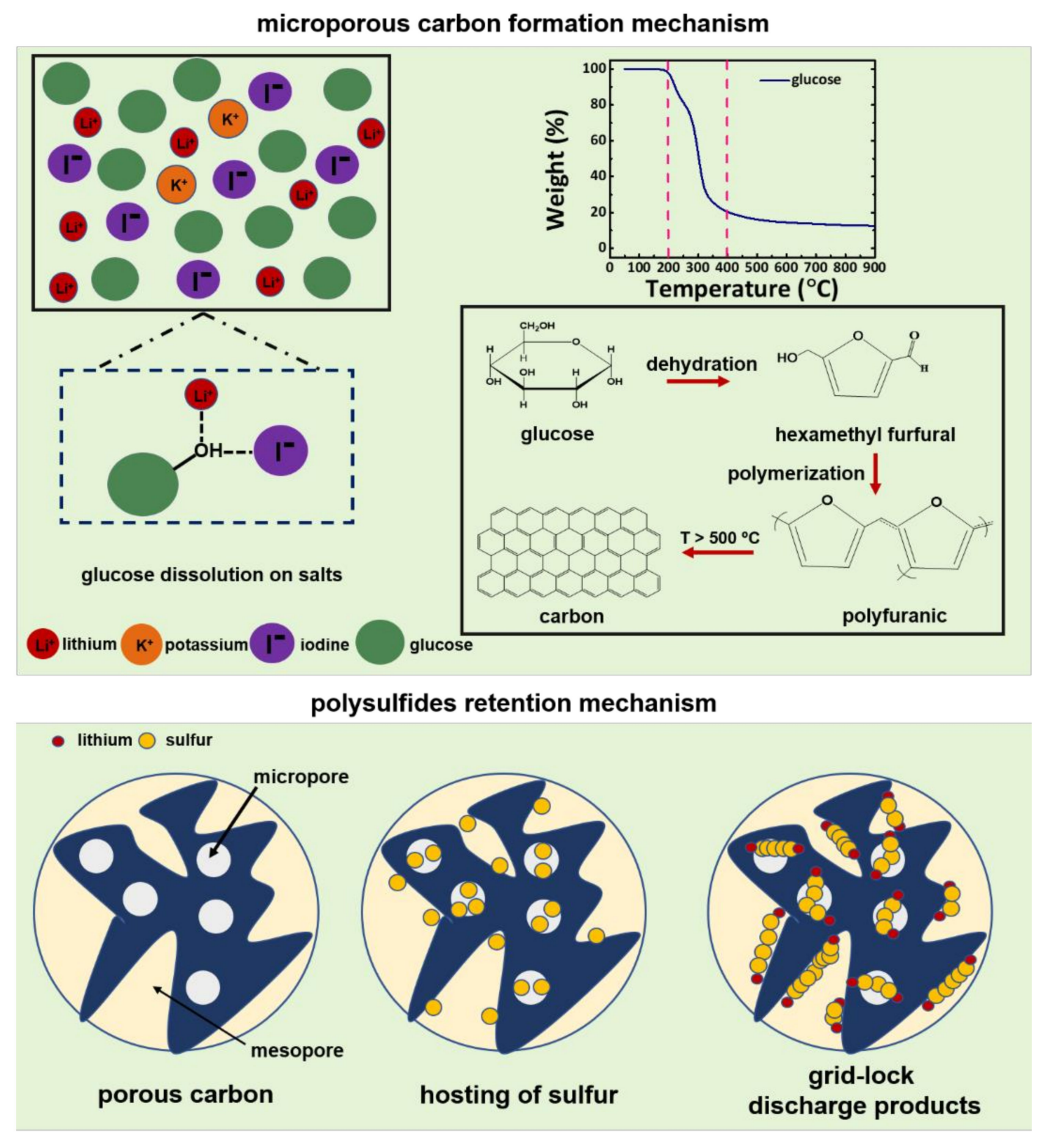
Formation of meso- and micro-porous carbon, hosting of sulfur, and the discharge product retention mechanism.

Glucose, as a biomass carbon source, was carbonized in the presence of the eutectic salt mixture of LiI and KI at 800 °C (Scheme 1). The melting temperature of the eutectic salt mixture of LiI/KI was about 275 °C; therefore, the polyaromatic condensation of glucose occurred in the liquid phase salt mixtures. The glucose carbonization process proceeded with dehydration, the formation of different intermediates, and polymerization of the intermediates [33]. Li salt assisted in the dissolution of glucose on the salt medium by generating a cation and anion interaction with the hydroxyl group of glucose. The action helped to disengage the glucose from an extensive hydrogen bond among the functional group. Additionally, the comparative broader radius and stronger nucleophilicity of the I anion stimulated the strength of the glucose’s solubilization on the salt medium. In the admixture, the K cation did not influence the ionic interaction with glucose; rather, it promoted the reduction of the melting temperature of the eutectic salt mixture, as well as its furnishing as a templating agent [34,35]. The overall progress served to create a new mode of intermediates before starting the polycondensation of carbon rather than pure glucose without salts. The presented strategy helped to form amazingly hierarchical porous carbonized materials using the high-temperature solvent method. Here, molten salts assisted in the construction of micropores on the surface of carbon materials with mesopores. Micropores promoted the hosting of sulfur on the structure. Subsequently, polysulfide discharge products were gridlocked on the system; thus, polysulfide sustained on the cathode structure will help minimize the dissolution on the electrolyte. Further, it will help to reduce the formation of parasitic mass to retain low internal resistance. At the same time, mesopores will maintain the lithium-ion transportation channel. Furthermore, a high surface area, good surface contact, and a high degree of aromatic condensation will support in attenuating local resistance.

**Figure 1:**
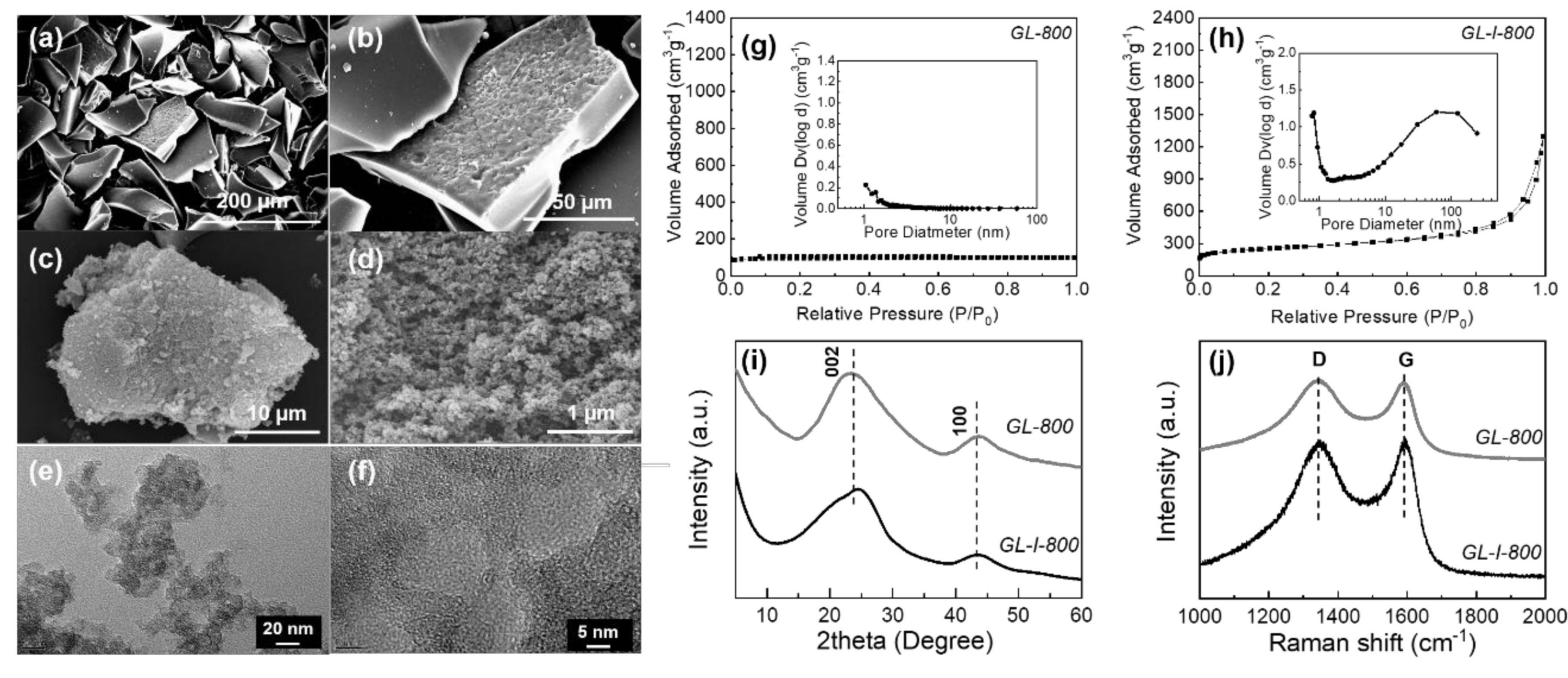
Scanning electron microscope (SEM) images of (**a**,**b**) GL-800 (glucose carbonized at 800 °C) and (**c**,**d**) GL-I-800 (glucose carbonized with eutectic salt LiI/KI at 800 °C). (**e**,**f**) Transmission electron microscope (TEM) images of GL-I-800. N_2_ adsorption–desorption isotherm of (**g**) GL-800 and (**h**) GL-I-800 (inset image is pore size distribution). (**i**) X-ray diffraction (XRD) patterns and (**j**) Raman spectra of GL-800 and GL-I-800.

The resulting samples’ morphology was analyzed by a scanning electron microscope (SEM) and transmission electron microscope (TEM). Figure 1c,d shows that the GL-I-800 particles have various shapes and sizes (from 10 to 40 µm). Carbonized glucose without eutectic salts shows a cube-like and nonporous surface (Figure 1a,b). The eutectic salts of LiI/KI help to create pores on the surface as well as the core of the structure arranged with a small gap between particles. The presence of the micropore channel with a continuous mesopore channel on the core inside the frame is observed. Figure 1d shows that the GL-I-800 particles have tiny nanoflakes with micropores and are interconnected by large-scale porous networks. There is a stark difference in the morphological scenario compared with the previous work using the LiBr/KBr salts to produce amorphous carbon, indicating that the LiI/KI solvent had different chemical interactions compared with the Br-based salt mixture [36]. In LiBr/KBr, the formation of the 3D porous microstructure, in contrast with LiI/KI, facilitated the formation of a particle shape with a porous structure and a mesoporous and macroporous pore route. In appearance, it does seem to be an agglomeration of nanoflakes by the eutectic salts of LiI/KI. Iodide has a larger anion size and higher nucleophilicity than bromide, which has a strong anion interaction with glucose and influences the firm dissolution of glucose. Therefore, it can be presumed that the formation of nanoflakes occurred rather than the curved three-dimensional porous structure from bromide. Additionally, empty spaces between nanoparticles are observed clearly in the TEM image in Figure 1e,f. It is reasonable to think that different morphologies between Br- and I-based materials originated from the usage of different anions, namely Br^−^ and I^−^. To further study the surface area and the porous structure of the carbonized materials, N_2_ adsorption–desorption analysis was performed. The N_2_ adsorption–desorption isotherm of the GL-I-800 shows a type III isotherm indicating the nonhomogeneous mesoporous systems (Figure 1h) [29]. The BET (Brunauer–Emmett–Teller) surface area and the pore volume of GL-I-800 are 844 m^2^ g^−1^ and 1.6 cm^3^ g^−1^, respectively. Conversely, the glucose’s carbonization without the eutectic salts of LiI/KI shows a surface area of 302 m^2^ g^−1^ and a pore volume of 0.15 cm^3^ g^−1^ (Figure 1g; Table 1). It is apprehensible that LiI/KI impacts the carbonization process by increasing the surface area and the pore volume of GL-I-800. The pore size distribution of GL-I-800 calculated by the nonlocal density functional theory (NLDFT) shows a hierarchical porous structure that contains micropores of ~0.8 nm, mesopores centered at 50 nm, and macropores of 50–200 nm. With macropores induced by eutectic salts, the constructed hierarchical porous structure of GL-I-800 offers a large interspace for variable intermediate species during the charge/discharge process of the Li-S battery. Remarkably, the total pore volume of GL-I-800 is superior to that of GL-Br-800 [37]. The carbonization trend occurs with the formation of some intermediates (Scheme 1). The dehydration of glucose is a key process to solvate the cations of the salt’s admixture. In the localized adjoining, water molecules occur on the cation’s inward coordination sphere, and the anion is kept free in the medium [38,39]. Hydrated molten salt and the cation interact with the electronegative oxygen atom and the anion with the hydrogen of the hydroxyl groups of the glucose. Furthermore, the ion–glucose interaction eases the dissolution by breaking the hydrogen bond among the glucose and the flow of glucose surrounding the molten salt [40,41]. The shape of the localized position can be compared to the thin coating of carbonaceous materials on the salt medium’s surface. This is a subtle and effective way to exfoliate or dissolve the carbonaceous materials to obtain a microporous porous hierarchical architecture [35]. The manner can be endorsed from the architectural differences in the GL-I-800 and GL-800 particles from the SEM results (Figure 1a,d).

As the polymerization process progressed, the formation of an *sp^2^* hybridized carbon domain was promoted. The solvated glucose product was diffused on the molten salt, followed by the ionic interaction between the salt’s ion and the *sp^2^* carbon edifice [42]. Consequently, such an ionic interaction imparts a large surface area and void space after the removal of salts. It is considered that, in terms of surface morphology, the larger ionic size of I^−^ compared to Br^−^, the interactions between carbonaceous materials and ions, as well as the effect of salt removal, are harsher for I-based materials than Br-based materials.

X-ray diffraction (XRD) was employed to investigate the crystal structure of the synthesized GL-I-800 samples. As shown, the crystal structure had a highly disordered microstructure similar to hard carbon. As shown in the XRD pattern in Figure 1i, the GL-I-800 sample contains a distinct diffraction peak at around 24.36° and at around 44.83°, which can be attributed to (002) for the interlayer space of the graphitic hexagonal ring and (100) for in-plane graphene facets of the carbonaceous materials with a high graphitization nature, respectively [20,22]. In the GL-800 sample, 002 and 100 peaks are identified at 23.34° and 44.39° (Figure 1i). The calculated interlayer space between the graphitic layer of GL-I-800 is about 0.37 nm, which shows a constricting trend compared to the pristine carbonized GL-800 sample of 0.38 nm. The calculated lateral sizes (*L*_a_) of the GL-I-800 and GL-800 samples are 3.63 and 2.96 nm (Table 2), respectively, which are substantially smaller values in comparison to the previously synthesized carbon materials using LiBr/KBr molten salts (*L_a_*; 4.4 nm) [36]. Here, broad and low-intensity peaks signify both the amorphous and short-range crystalline phases present together. It is hard to extract precise structural analysis. We have to depend or assume semiquantitative analysis based on the peaks shape, position, and intensity for the structural characterization. Crystallite size (*L*_c_) increased for the eutectic salt-treated GL-I-800 sample to 0.88 nm from 0.81 nm for the GL-800 sample. The calculated numerical stacking numbers of the graphitic layer for GL-I-800 and GL-800 were 3.41 and 3.13. The graphite crystallite portion could be assumed from the *R*-value, which can be calculated from the ratio of the observed peak intensity to the background peak intensity [43]. The *R*-value has a rising trend for the GL-I-800 sample compared to the GL-800 sample, from 2.3 to 2.36 (Table 2). The phenomenon could be explained due to the significant amount of graphite-like structure formation rising in the high-temperature solvent medium, facilitating further aromatic condensation.

Raman spectroscopy was carried out to study the crystallography and structure of the carbon material in the GL-I-800 sample. As shown in Figure 1j, distinct D and G bands appeared in the Raman spectra at 1350; 1580 cm^−1^ corresponds to the vibration of *sp^3^* domains due to the defects in the vicinity of the carbon structure, such as C-O and C=O, and the vibration of the *sp^2^*-hybridized carbon atom, respectively. Typically, it is possible to assess the aromatic structural disorder from the intensity of the G to D ratio (*I*_G_/*I*_D_). The higher the *I*_G_/*I*_D_ ratio, the more structural in-plane aromatic length disturbance exists. The intensity ratios of *I*_G_/*I*_D_ were found to be 1.016, indicating substantial crystallite components in the microstructure of the GL-I-800 sample. On the contrary, the carbonized GL-800 (*I*_G_/*I*_D_: 0.99) sample shows a higher disorder than the GL-I-800 (*I*_G_/*I*_D_: 1.02) sample. It can be assumed that the different salts’ anion or cation interactions with carbon intermediates have an individual impact on the structural configuration arrangement [39]. Here, Raman’s outcome is well-matched with the earlier calculated in-plane length of the carbon from the XRD analysis (Table 2).

**Figure 2:**
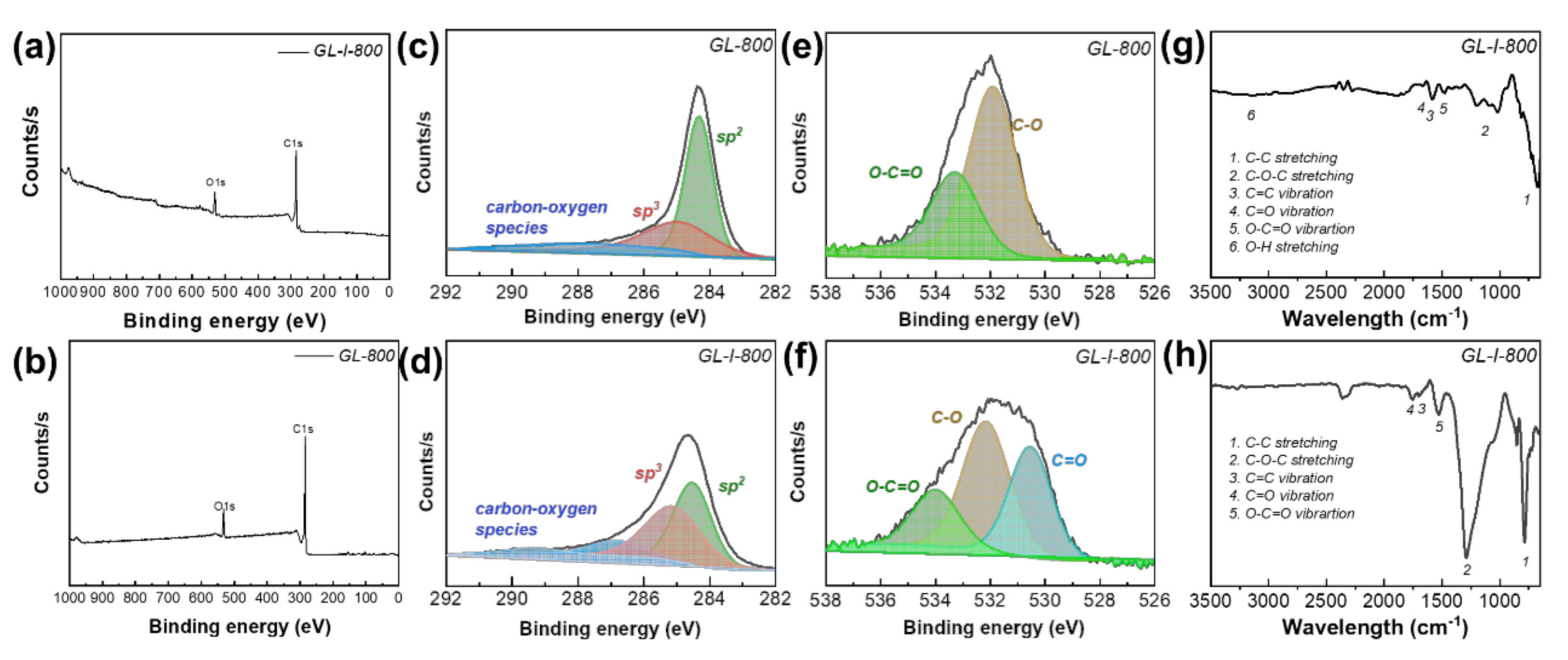
X-ray photoelectron spectroscopy (XPS) survey spectra of (**a**) GL-800 and (**b**) GL-I-800. Deconvolution of the high-resolution XPS spectra of GL-800 and GL-I-800: (**c**,**d**) C 1s and (**e**,**f**) O 1s. FT-IR spectra of (**g**) GL-800 and (**h**) GL-I-800.

**Figure 3:**
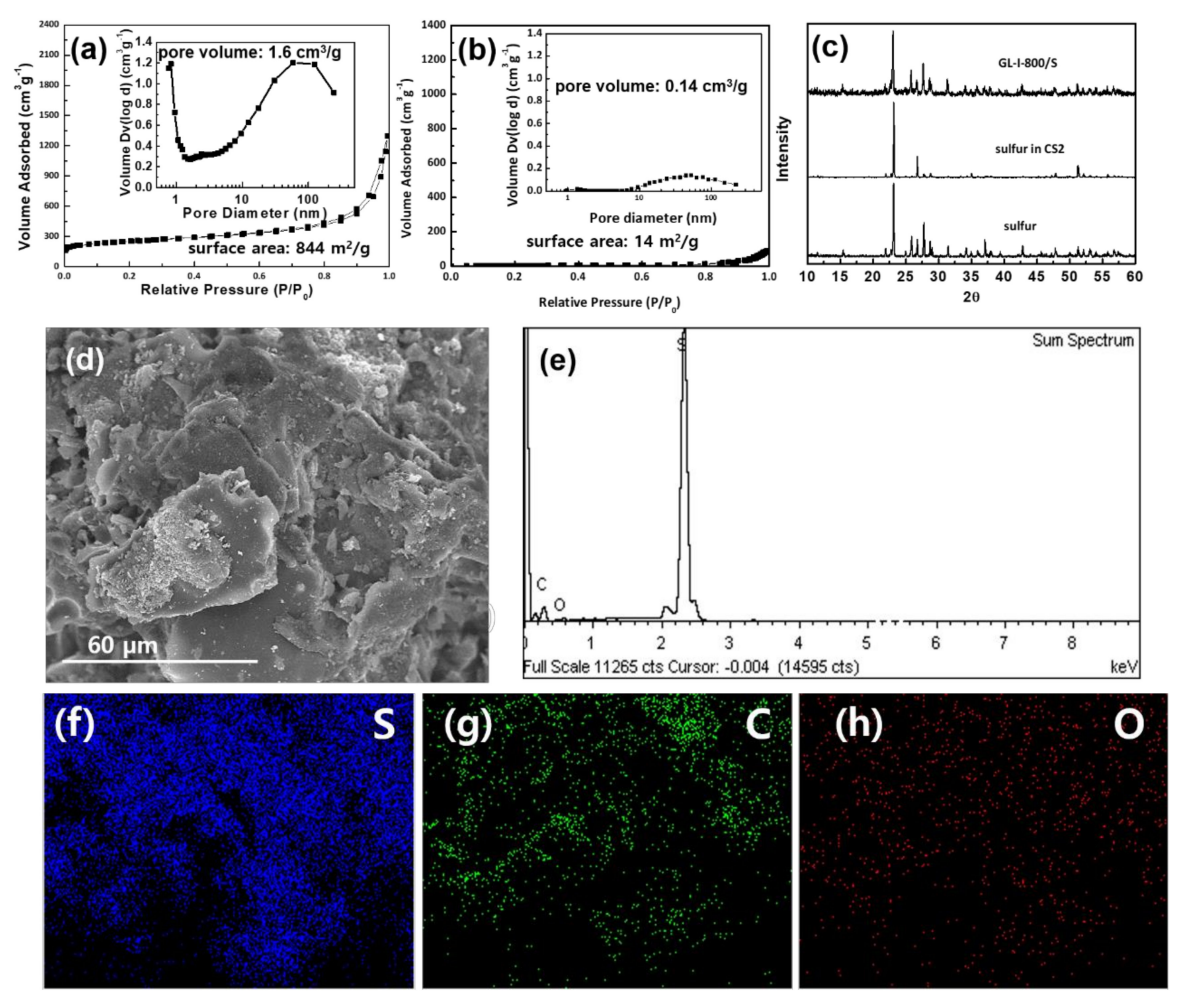
N_2_ adsorption–desorption isotherm of (**a**) GL-I-800 and (**b**) GL-I-800+S (inset images are pore size distribution). (**c**) XRD patterns of sulfur, sulfur recovered from CS_2_ solution, and sulfur infiltrated GL-I-800. (**d**) SEM image of GL-I-800+S. (**e**) Energy-dispersive spectroscopy (EDS) spectra of GL-I-800+S. EDS mapping of GL-I-800+S: (**f**) sulfur; (**g**) carbon; (**h**) oxygen.

To grasp the structural chemical status of carbonized materials, X-ray photoelectron spectroscopy (XPS) and Fourier transform infrared spectroscopy (FT-IR) analysis were performed (Figure 2). A significant presence of oxygen functional groups is on the carbonized materials (Figure 2a,b). In general, XPS depicts a clear picture of the surface chemical anatomy. A substantial portion of the presence of *sp^3^* carbon defects along with the carbon–oxygen species is on the GL-I-800 sample rather than the GL-800 sample (Figure 2c,d) [44]. It can be presumed this phenomenon is due to the salt conditions, exfoliation of the surface carbon, and the iodine impact on the glucose [34,35]. The expanded exposed surface of the GL-I-800 sample compared to the GL-800 sample provides better *sp^3^* detection, which can be well-matched with the SEM, surface area, and pore volume results. It is outstanding to observe that the appearance of the C=O functional group in the GL-I-800 sample (Figure 3e,f) is beneficial to the higher adsorption bond strength on the polysulfides, which is also confirmed from the FT-IR spectra (Figure 3g,h) [45].

Following this, the presence of polar oxygen on the amorphous carbon structure will significantly impact the lessening of the dissolution of the polysulfides on the electrolytes (Table 3). XPS and FT-IR analyses confirmed the presence of C-O and C=O’s significant defects, which will promote polar adsorption of polysulfides on the carbon surface. As a consequence, it will improve the ultimate performance of the LSB. In accordance with the above interpretation, GL-I-800 has a well-crystallized structure in a short range with a highly polar surface oxygen functional group compared with GL-800.

As a sulfur-supporting material, GL-I-800 has a high pore volume structure. This is productive regarding high-loading sulfur infiltration into micropores, which enables these additional reduction reactions into mesopores. We performed BET, XRD, and electron-dispersive X-ray spectroscopy (EDX) mapping before and after sulfur infiltration to design an accurate mass of sulfur loading in the cathode. Figure 3a shows that the BET surface area and pore volume of GL-I-800 are 816 m^2^ g^−1^ and 1.6 cm^3^ g^−1^, respectively. After sulfur infiltration using CS_2_ solution, the BET surface area and pore volume in Figure 3b dropped from 816 m^2^ g^−1^ to 14 m^2^ g^−1^ and 1.5 cm^3^ g^−1^ to 0.14 cm^3^ g^−1^, respectively, indicating that most of the pores were filled with elemental sulfur via melt-diffusion at 150 °C. Based on the difference in pore volume (0.122 cm^3^) before and after the sulfur infiltration of GL-I-800 at 80 mg and a density of S (2 g cm^−3^), it is estimated that the moderate sulfur loading value is 3 g S for g^−1^ GL-I-800. Furthermore, we composed the cathode above 2 mg for GL-I-800 cm^−2^ and high sulfur infiltration into GL-I-800 around >6 mg S cm^−2^. Significantly, one of the most important aspects is the high areal capacity of LSBs comparable to that of the LIBs (4 mAh cm^−2^). High-loading sulfur is essential for rival LIBs [17]. The powder X-ray diffraction (XRD) patterns of sulfur, sulfur in CS_2_, and the GL-I-800+S composite are displayed in Figure 3c. After loading the sulfur into the GL-I-800 host materials, the XRD patterns of the resultant GL-I-800+S composite reveal that the elemental sulfur of S_8_ is well maintained during the preparation of the electrode. Crystalline sulfur is maintained in the XRD patterns of Gl-I-800+S after sulfur infiltration, indicating residual elemental sulfur is placed on the carbon particle’s surface (Figure 3c). The EDS elemental mapping also confirmed the successful infiltration of sulfur, as shown in Figure 3d–h. From the figure, it is shown that elemental sulfur is uniformly distributed in the Gl-I-800+S composite.

**Figure 4:**
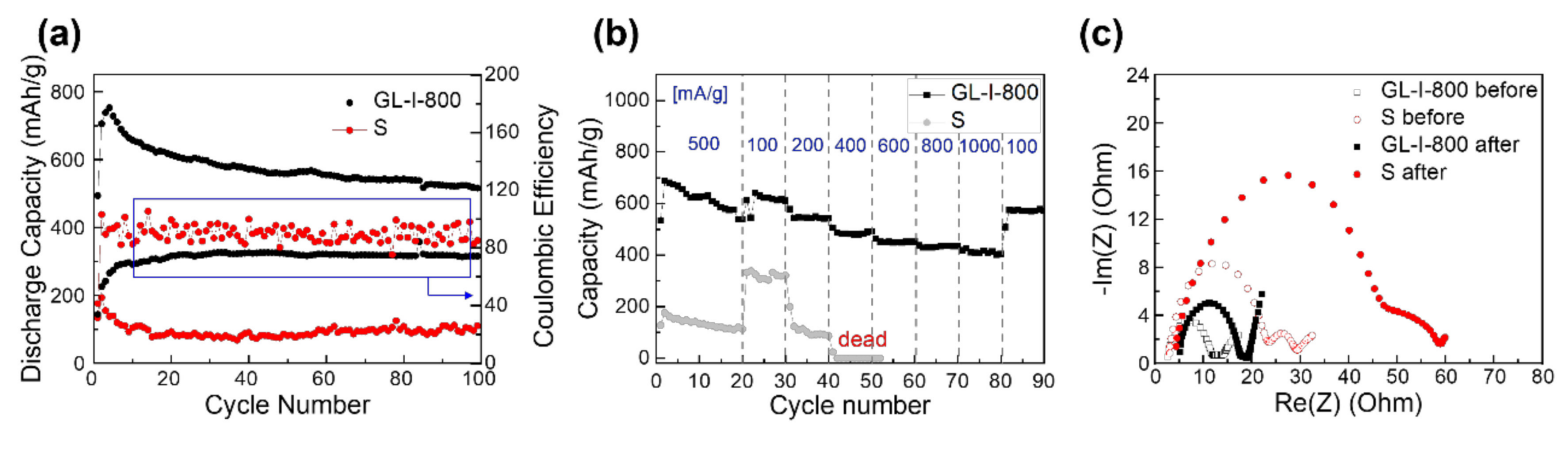
Electrochemical performance of Gl-I-800+S and pristine S: (**a**) Discharge capacity vs. cycle number, (**b**) rate capability, and (**c**) electrochemical impedance spectroscopy (EIS) spectra.

To investigate the electrochemical performance of GL-I-800 as a sulfur host material, cyclic voltammetry (CV), galvanostatic cycling with potential limitation (GCPL), and electrochemical impedance spectroscopy (EIS) measurements were carried out and compared with the bulk sulfur cathode including only the conductive carbon additive Super P (Figure 4). We used a solution impregnation method, which enabled a high degree of the dispersion of sulfur into the carbon matrix and thereby improved the electrochemical behavior of GL-I-800 toward sulfur. Solid elemental sulfur (S_8_) dissolved in a carbon disulfide was impregnated into the GL-I-800, which was induced by the capillary drive force exerted by the micropores/mesopores. Consequently, we were able to obtain a sulfur content of 53 wt % in the GL-I-800+S composite. Figure 4a shows the cycling performance of GL-I-800+S and the bulk sulfur cathode at a constant current density of 500 mA g^−1^ with an areal sulfur loading mass of 2 mg S/cm^2^, which exhibits an enhanced initial discharge capacity of 780 mAh g^−1^ with a moderate capacity loss per cycle of 0.36% for 100 cycles. The specific discharge capacity of GL-I-800+S yields 516 mAh g^−1^ after 100 cycles, whereas bulk S exhibits only 110 mAh g^−1^. Benefiting from the high utilization ratio of sulfur, the GL-I-800+S cathode delivered a high discharge capacity after 100 cycles [46]. This superior performance indicates that the porous carbon matrix structure of the GL-I-800+S host provides abundant anchoring sites to suppress polysulfide shuttling, further retaining soluble sulfur species and improving the discharge capacity. The kinetic rate performance of the GL-I-800+S cathode and bulk sulfur was investigated under various rates with a galvanostatic mode to illustrate the structural advantages of the porous carbon matrix (Figure 4b). First, the cells’ pre-electrochemical activation was carried out at a current density of 500 mA g^−1^ followed by cycling at a current density of 100, 200, 400, 600, 800, 1000, and 100 mA g^−1^ in sequence. Impressively, when the current density was abruptly switched back to 100 mA g^−1^, the reversible discharge capacity of GL-I-800+S was almost restored without abrupt capacity degradation.

On the other hand, the cells with a bulk sulfur cathode precluded at 200–400 mA g^−1^, signifying that the bulk sulfur cathode is vulnerable to high current density. The electrochemical impedance spectroscopy (EIS) study was further conducted for GL-I-800+S and the bulk sulfur cathode before and after cycling. GL-I-800+S and bulk sulfur showed increased resistance after cycling, where the values of *R*_ion_ (ionic resistance) and *R*_ct_ (resistance to charge transfer) of the GL-I-800+S cathode were overwhelmingly smaller than those of the bulk sulfur electrode. This indicates a smaller shuttling effect for the dissolution of polysulfides for the GL-I-800+S cathode than the bulk sulfur electrode (Figure 4c; Table 4) [47].

**Figure 5:**
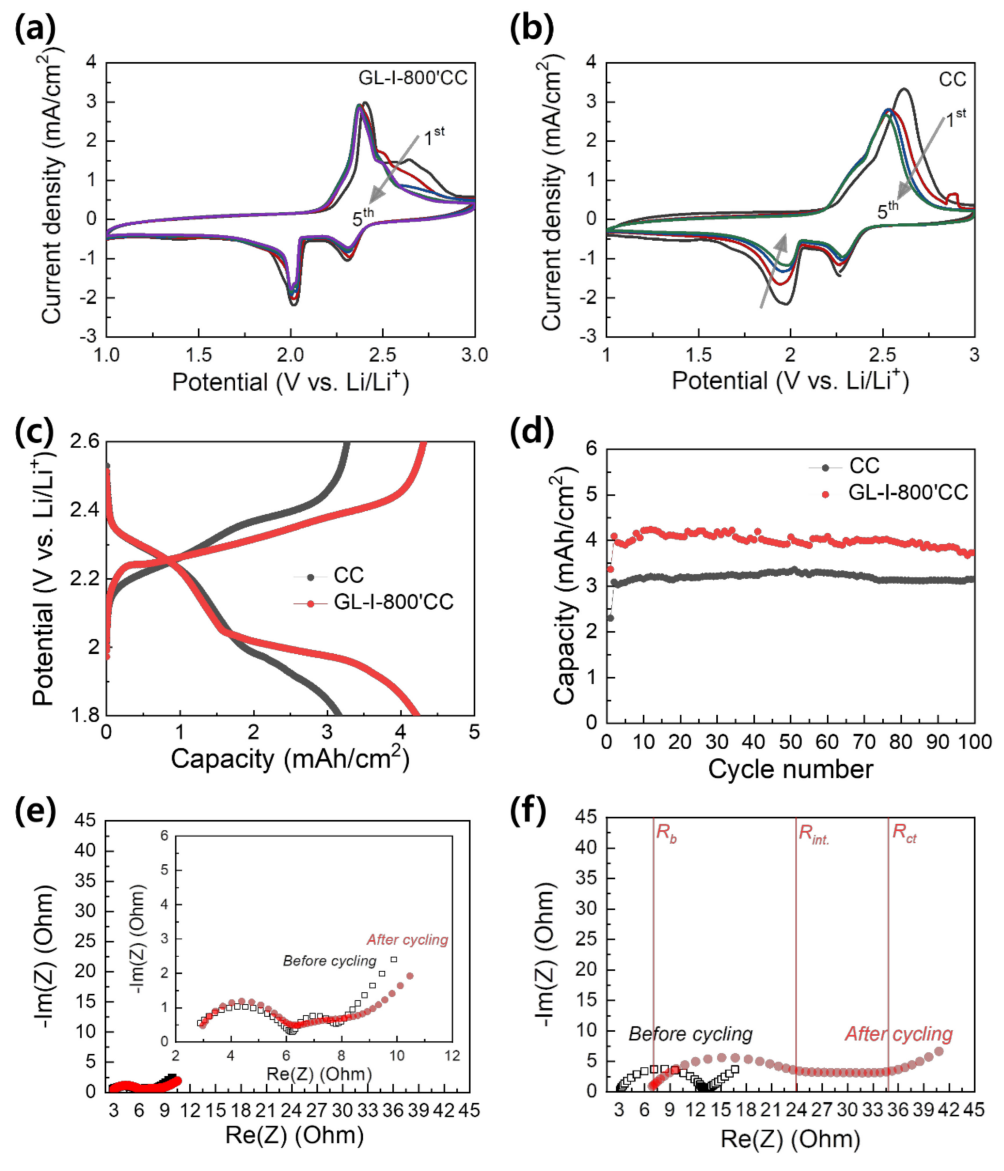
Cyclic voltammetry (CV) diagram of (**a**) Gl-I-800′CC and (**b**) CC. (**c**) The second galvanostatic cycling with potential limitation (GCPL) profile at 500 mA/g. (**d**) Capacity vs. cycle number at 500 mA/g. Electrochemical impedance spectra (EIS) of (**e**) GL-I-800′CC and (**f**) CC.

To evaluate the superiority of GL-I-800 under high sulfur loading conditions (6 mg S cm^−2^), GL-I-800 was implemented into the commercial activated carbon-based current collector, i.e., carbon cloth (CC), as an additional sulfur host (Figure 5). The effect of GL-I-800’CC on redox reactions was first investigated by cyclic voltammetry (CV) between a potential window of 1.0–3.0 V vs. Li/Li^+^ at a scan rate of 0.1 mV s^−1^. It can be seen that the second cathodic peak is generated at ~2.05 V vs. Li/Li^+^ in GL-I-800’CC with negligible current degradation, whereas that of CC is shown below 2.0 V vs. Li/Li^+^ with the significantly decreased current density from −3 to −1.8 mA cm^−2^ after five cycles (Figure 5a,b). The same trend of decreased polarization without a current loss was also observed in the anodic peak in the range between 2.2 and 2.5 V vs. Li/Li^+^. The increased current density of the second cathodic peak in CV translates into greater capacity generation from the reduction reaction of high polysulfides to low polysulfides or lithium sulfide, which matches well with the increase (~1 mAh cm^−2^) in the capacity generation from the second plateau of the GCPL profile for GL-I-800’CC (Figure 5c,d) [48]. The improved electrochemical behavior toward sulfur in the presence of GL-I-800 persists for 100 cycles, which is enabled by the high-efficiency reversible reaction between sulfur and lithium sulfide (Figure 5d,f). In order to investigate the electrochemical behaviors and kinetics of LSBs during a specific cycle, the impedance spectra were recorded in the frequency range between 50 mHz and 500 kHz with an AC potential difference with an amplitude of 5 mV. As shown in Figure 5e,f, the Nyquist plots consist of a semicircle in the HF region (500 kHz–1 kHz), a semicircle in the MF region (1 kHz–1 Hz), and a sloping line in the LF region (1 Hz–50 mHz). Two semicircles are hardly distinguished from each other due to their overlap. To get a better understanding of impedance parameters, the Nyquist plots were fitted with a constant phase element (CPE) model for the rough and porous electrode surface showing the nonideal behavior. This extracts the information of bulk cell resistance (*R*_b_) related to the electrolyte, interphase contact resistance (*R*_int._) related to the process of electron conduction from the current collector to the reaction site, charge-transfer resistance (*R*_ct_) related to the process of charge transfer between the conductive agent and the electrolyte, and Warburg impedance (*R*_w_) related to Li-ion diffusion. These values are summarized in Table 5. After cycling, the diameter of the first semicircle, corresponding to interphase contact resistance, decreases from 3.9 to 3.0 Ohm for GL-I-800’CC and from 8.6 to 2.1 Ohm for CC because of electrochemical activation during repeated cycling. On the other hand, the second semicircle, corresponding to charge-transfer resistance, shows minor increases from 1.2 to 3.2 Ohm for GL-I-800′CC as compared to that of CC.

Table 6 compares the electrochemical performance of this work with some of the other studies. In the present study, the eutectic salt-based LiI/KI-synthesized microporous carbon (516 mAh g^−1^, at 500 mA g^−1^, 100 cycles) showed improved performance compared to the composite of CNT@ porous carbon (derived from Al-based metal–organic framework) (424 mAh g^−1^, at 500 mA g^−1^, 100 cycles) [24]. Additionally, GL-I-800/S shows an improved retention capacity (66%) than the CNT/porous carbon/S (60%). The present study presents an opportunity to further improve performance by tuning pore and structural engineering in the future. Different halide anion implementations and precursor-to-salt ratio adjustments could be possible routes to progress further.

## 4. Conclusions

A straightforward strategy has been taken to synthesize a unique hierarchical porous carbon structure. The most attractive role is the LiI/KI eutectic salts mixture, which delivered a high-temperature solvent medium for the carbonization of glucose. Cation lithium and iodide assisted in dissolute the glucose. Furthermore, the larger anion size and high nucleophilicity of iodide impacted the formation of a different mode of intermediates before the start of polycondensation. It ultimately provided a higher surface area (844 m^2^ g^−1^) and an increased pore volume (1.6 cm^3^ g^−1^) of microporous and mesoporous carbon. Consequently, greater sulfur-hosting capabilities and micropores assisted in gridlocking the polysulfides on the cathode, which improved the initial capacity of the GL-I-800+S cathode to 780 mAh g^−1^ at a current density of 500 mA g^−1^ with a mere 0.36% capacity loss per cycle for 100 cycles. The exposed modality showed a significant improvement in the next-generation energy storage system of lithium–sulfur batteries. Finally, this finding presents a new portal of scope to further the research progress.

## Figures and Tables

**Table 1:** The comparison of surface area and pore volume of GL-800 (glucose carbonized at 800 °C) and GL-I-800 (glucose carbonized with eutectic salt LiI/KI at 800 °C).

Materials	Surface Area (m^2^ g^−1^)	Pore Volume (cm^3^ g^−1^)
GL-800	302	0.15
GL-I-800	844	1.60

**Table 2:** Crystal structure of GL-800 and GL-I-800: interlayer space, *L*_c_, *L*_a_, stacking number, *R* factor, and *I*_G_/*I*_D_ among.

Materials	*d*_002_ (nm)	*d*_100_ (nm)	*L*_c_ (nm)	*L*_a_ (nm)	Stacking Number	*R* factor	*I*_G_/*I*_D_
GL-800	0.4	0.2	0.8	3.0	3.1	2.3	0.99
GL-I-800	0.4	0.2	0.9	3.6	3.4	2.4	1.02

**Table 3:** Elemental analysis from X-ray photoelectron spectroscopy (XPS).

Materials	Atomic Percent (at %)		Ratio
	C	O	O/C
GL-800	78.86	21.14	0.27
GL-I-800	74.13	25.87	0.35

**Table 4:** Electrochemical impedance spectra (EIS) fitting results using a constant phase element (CPE) model.

Electrode Materials	*R*_b_ (Ω)	*R*_int._ (Ω)	*R*_ct_ (Ω)	Warburg (Ω^0.5^)
Bulk S_Before	3.6	20.2	7.8	1.9
Bulk S_After	5.8	32.2	13.0	2.0
GL-I-800_Before	2.8	0.2	10.0	2.1
GL-I-800_After	6.4	14.8	14.9	3.3

**Table 5:** EIS fitting results using a CPE model.

Electrode Materials	*R*_b_ (Ω)	*R*_int._ (Ω)	*R*_ct_ (Ω)	Warburg (Ω^0.5^)
GL-I-800′CC_Before	2.354	3.934	1.187	1.348
GL-I-800′CC_After	2.698	3.053	3.232	0.982
CC_Before	2.562	8.574	3.539	1.632
CC_After	6.448	2.137	15.61	1.923

**Table 6:** The comparison of electrochemical performance.

No.	Sample	Current Density (mA g^−1^)	Capacity (mAh g^−1^)	Cycle	Sulfur Loading (mg cm^−2^)	Reference
1	Ordered bimodal mesoporous carbon/60 wt % S	1675	550	100	-	[19]
2	Graphene/70 wt % S	1675	615	100	-	[20]
3	CNT@ZnS/S	335	500	500	3.1	[23]
4	CNT@ porous carbon /50 wt % S	500	424	100	-	[24]
5	Ordered mesoporous carbon/50 wt % S	1675	730	100	-	[25]
6	S@CNT/C-NC	837	586	500	-	[26]
7	Ni/PCNFO (porous carbon nano fibers)/S	600	800	40	4.4	[27]
8	Graphene/S	335	840	100	0.86	[29]
9	Microporous carbon/S	500	516	100	3	This work
